# Distinct cortical locations for integration of audiovisual speech and the McGurk effect

**DOI:** 10.3389/fpsyg.2014.00534

**Published:** 2014-06-02

**Authors:** Laura C. Erickson, Brandon A. Zielinski, Jennifer E. V. Zielinski, Guoying Liu, Peter E. Turkeltaub, Amber M. Leaver, Josef P. Rauschecker

**Affiliations:** ^1^Department of Neuroscience, Georgetown University Medical Center, WashingtonDC, USA; ^2^Department of Neurology, Georgetown University Medical Center, WashingtonDC, USA; ^3^Department of Physiology and Biophysics, Georgetown University Medical Center, WashingtonDC, USA; ^4^Departments of Pediatrics and Neurology, Division of Child Neurology, University of Utah, Salt Lake CityUT, USA; ^5^National Institutes of Health, BethesdaMD, USA; ^6^MedStar National Rehabilitation Hospital, WashingtonDC, USA; ^7^Department of Neurology, University of California Los Angeles, Los AngelesCA, USA

**Keywords:** McGurk effect, superior temporal sulcus, dorsal stream, sensorimotor, cross-modal, multisensory, speech

## Abstract

Audiovisual (AV) speech integration is often studied using the McGurk effect, where the combination of specific incongruent auditory and visual speech cues produces the perception of a third illusory speech percept. Recently, several studies have implicated the posterior superior temporal sulcus (pSTS) in the McGurk effect; however, the exact roles of the pSTS and other brain areas in “correcting” differing AV sensory inputs remain unclear. Using functional magnetic resonance imaging (fMRI) in ten participants, we aimed to isolate brain areas specifically involved in processing congruent AV speech and the McGurk effect. Speech stimuli were composed of sounds and/or videos of consonant–vowel tokens resulting in four stimulus classes: congruent AV speech (AV_Cong_), incongruent AV speech resulting in the McGurk effect (AV_McGurk_), acoustic-only speech (A_O_), and visual-only speech (V_O_). In group- and single-subject analyses, left pSTS exhibited significantly greater fMRI signal for congruent AV speech (i.e., AV_Cong_ trials) than for both A_O_ and V_O_ trials. Right superior temporal gyrus, medial prefrontal cortex, and cerebellum were also identified. For McGurk speech (i.e., AV_McGurk_ trials), two clusters in the left posterior superior temporal gyrus (pSTG), just posterior to Heschl’s gyrus or on its border, exhibited greater fMRI signal than both A_O_ and V_O_ trials. We propose that while some brain areas, such as left pSTS, may be more critical for the *integration* of AV speech, other areas, such as left pSTG, may generate the “corrected” or merged percept arising from conflicting auditory and visual cues (i.e., as in the McGurk effect). These findings are consistent with the concept that posterior superior temporal areas represent part of a “dorsal auditory stream,” which is involved in multisensory integration, sensorimotor control, and optimal state estimation (Rauschecker and Scott, 2009).

## INTRODUCTION

Two distinct sensory signals are seamlessly integrated during typical speech processing: sounds and facial movements. The integration of acoustic and visual speech cues is frequently studied using the McGurk effect (McGurk and MacDonald, 1976), wherein sounds and facial movements are deliberately mismatched to elicit the perception of an entirely different and illusory consonant–vowel (CV) token. One common example is when the sound “ba” is dubbed onto the visual articulation of “ga,” an illusory bimodal “McGurk” percept of “da” results. Yet, the precise neural mechanisms governing integration of congruent audiovisual (AV) speech signals and the subtle perceptual shift of the McGurk effect remain unclear.

Numerous neuroimaging (Sams et al., 1991; Jones and Callan, 2003; Sekiyama et al., 2003; Skipper et al., 2007; Bernstein et al., 2008; Benoit et al., 2010; Wiersinga-Post et al., 2010; Irwin et al., 2011; Nath et al., 2011; Nath and Beauchamp, 2012; Szycik et al., 2012) and behavioral studies (Green et al., 1991; Green and Norrix, 1997; Tiippana et al., 2004, 2011; Nahorna et al., 2012) of the McGurk effect have been published, as well as one transcranial magnetic stimulation (TMS) study (Beauchamp et al., 2010). Substantial emphasis has been placed on the importance of the posterior superior temporal cortex (pST), specifically the left posterior superior temporal sulcus (pSTS), in the McGurk effect (Sekiyama et al., 2003; Bernstein et al., 2008; Beauchamp et al., 2010; Benoit et al., 2010; Irwin et al., 2011; Nath et al., 2011; Nath and Beauchamp, 2012; Szycik et al., 2012). However, other brain regions have also been linked to processing McGurk-type stimuli, including frontal (Skipper et al., 2007; Benoit et al., 2010; Irwin et al., 2011), insular (Skipper et al., 2007; Benoit et al., 2010; Szycik et al., 2012), and parietal areas (Jones and Callan, 2003; Skipper et al., 2007; Benoit et al., 2010; Wiersinga-Post et al., 2010), as well as other regions (Skipper et al., 2007; Bernstein et al., 2008; Wiersinga-Post et al., 2010; Nath et al., 2011; Szycik et al., 2012). While these experiments examine neural processes related to the McGurk effect, the precise role of each brain region implicated in the McGurk effect, particularly within the pST, is still not completely understood.

The neuroanatomical variability associated with the McGurk effect may be explained by variations in experimental design, as well as differing analytical approaches. Previous studies have probed the McGurk effect using a variety of statistical approaches. Examples include direct contrasts between incongruent McGurk speech versus congruent AV speech (Jones and Callan, 2003; Skipper et al., 2007; Bernstein et al., 2008; Benoit et al., 2010; Irwin et al., 2011; Szycik et al., 2012), or correlations between functional magnetic resonance imaging (fMRI) BOLD activity and McGurk percept reports/susceptibility (Benoit et al., 2010; Wiersinga-Post et al., 2010; Nath et al., 2011; Nath and Beauchamp, 2012). However, these approaches do not isolate regions specifically sensitive to AV signals versus unimodal signals, where interactions of auditory and visual sensory input are likely to occur. This suggests that other methods may be needed to further evaluate the neural correlates of the McGurk effect. Others (Calvert and Thesen, 2004; Beauchamp, 2005b; Laurienti et al., 2005; Stein and Stanford, 2008; Goebel and van Atteveldt, 2009) have discussed several ways to statistically identify neural correlates of multisensory integration, such as assessing the conjunction of auditory and visual signals, and examining differential activation magnitude between AV and unimodal signals (max criterion or super-additive approaches). Beauchamp (2005b) specifically showed that application of different statistical contrasts for AV signals compared to unimodal signals affected activation patterns in the temporal lobe, which is highly relevant when examining the neural correlates of the McGurk effect. Thus, the use of a different statistical approach may help to parse out the cortical processing mechanisms behind the McGurk phenomenon.

In the current study, we attempted to tease apart the distinct neural correlates involved in AV processing of congruent AV speech and McGurk speech. In ten participants using fMRI across the whole brain, we chose the max criterion (Beauchamp, 2005b), which identifies AV-processing regions that respond more strongly to AV stimuli relative to both unimodal auditory and visual stimulation alone. This approach allowed us to focus on brain areas optimized specifically for processing bimodal AV speech, rather than those that respond equally well or indiscriminately to bimodal AV and unimodal stimuli. We suggest that this method allowed for the isolation of AV-processing regions most likely to be involved in processing congruent AV speech or the change in perception accompanying the McGurk effect. This statistical approach has been successfully utilized to isolate AV-processing regions in several language studies (van Atteveldt et al., 2004, 2007; Szycik et al., 2008; Barros-Loscertales et al., 2013) and other types of AV studies (Beauchamp, 2005b; Hein et al., 2007; Watson et al., 2014). Since others have raised the issue of high individual anatomical/functional variability concerning the multisensory portion of the STS (Beauchamp et al., 2010; Nath and Beauchamp, 2012), we confirmed our group results in single-subject analyses, accounting for individual differences in gyral anatomy (Geschwind and Levitsky, 1968) and functional localization within pST. We sought to ensure the location of AV function relative to posterior superior temporal gyrus (pSTG), pSTS, and other landmarks within the pST. Distinguishing between the neural correlates related to AV processing of congruent AV speech and AV processing specific to perceptual ambiguity may help to extend ideas of multisensory functions within current sensorimotor models of language (Skipper et al., 2007; Rauschecker and Scott, 2009; Rauschecker, 2011).

## MATERIALS AND METHODS

### PARTICIPANTS

Ten volunteers (6 females; mean age = 25.72 years, SD = 3.01) contributed data to this study and were consented in accordance with Georgetown University Institutional Review Board. All participants were right-handed, and primary English speakers. Subjects were recruited through advertisement. Telephone screening ensured that all subjects were in good health with no history of neurological disorders, and reported normal hearing and normal or corrected-to-normal vision. Data from all ten participants were used in statistical analysis.

### CONSONANT–VOWEL (CV) TOKEN STIMULI

The following American-English CV tokens were recorded and digitized with sound from six volunteers (3 females and 3 males) articulating the following speech sounds: “ba,” “ga,” “pa,” and “ka,” using a Panasonic video-recorder and SGI O2 workstation. Audio and video tracks were edited and recombined using Adobe Premiere. In the videos, only the lower half of each speaker’s face was visible, minimizing the influence of gaze and facial processing. Four gain-normalized CV token stimulus types of 2 s duration were created for this experiment: 24 acoustic stimuli with the video track removed (unimodal auditory, A_O_), 24 video stimuli with the auditory track removed (unimodal visual, V_O_), 24 congruent AV stimuli (AV_Cong_), and 12 incongruent AV McGurk stimuli (AV_McGurk_). The relatively large number of different stimuli from six separate speakers for each stimulus type (AV_Cong_, AV_McGurk_, A_O_, V_O_) helped to reduce potential repetition effects. A_O_ stimuli contained only CV token sounds with no video display of corresponding lower facial movements; only a blank screen was shown. V_O_ stimuli contained a silent video display of lower facial movements during articulation of a CV token with no corresponding sound presented. AV_Cong_ stimuli contained sound and video from the original CV token recording. For example, auditory “ba” and visual “ba” were recorded from the same speaker during congruent, typical AV speech. AV_McGurk_ stimuli were created from combinations of differing sound and video CV token stimuli to produce two robust McGurk illusions (McGurk and MacDonald, 1976; Green et al., 1991; Green and Norrix, 1997). Twelve different McGurk stimuli were produced to reduce potential repetition effects, where each AV_McGurk_ stimulus was created from the same speaker and presented synchronously. The first set of McGurk stimuli consisted of sound “ba” dubbed onto a video of lips articulating “ga,” yielding six stimuli conveying the fused perception “da,” one for each recorded speaker. The second set of McGurk stimuli consisted of “pa” audio dubbed onto a video of lips articulating “ka,” producing six stimuli with the fused perception of “ta,” one for each recorded speaker.

### fMRI EXPERIMENT AND PARADIGM

Scans were acquired using a blocked design in a single fMRI session composed of two runs. AV_Cong_ blocks of trials were presented in the first run, and AV_McGurk_ blocks of trials were presented in the second run. A_O_ and V_O_ blocks of trial types were presented in both runs. Three block types were presented in a repeated “A–B–A–C” pattern as follows: AV, V_O_, AV, A_O_. Each block of trials contained only one type of stimuli, i.e., AV, V_O_, or A_O_. During each block, seven trials of stimuli (AV, A_O_, or V_O_) were presented continuously and pseudo-randomly at approximately every 2 s. For each stimulus block, two echo-planar imaging (EPI, or “functional”) volumes were collected, and the beginning of each EPI volume was separated by 6.5 s. CV token stimuli were 2 s in length. Thus, in order to create a 13 s stimulus block, actual presentation time for any single stimulus was fractionally less than 2 s. At the beginning of each run, three pre-stimulus “dummy” volumes were collected and removed before statistical analysis to allow for steady-state relaxation. Within each run, 20 blocks were presented, and 40 EPI volumes were acquired, consisting of 20 AV, 10 A_O_, and 10 V_O_ volumes. The total number of EPI volumes collected for both AV_Cong_ and AV_McGurk_ runs included: 20 AV_Cong_, 20 AV_McGurk_, 20 A_O_, and 20 V_O_.

In the MR scanner, binaural auditory stimuli were presented using a custom air-conduction sound system with silicone-cushioned headphones (Resonance Technologies, Van Nuys, CA, USA). The level of auditory stimuli was approximately 75–80 dB SPL, assessed using a B&K Precision Sound Level Meter. Videos (visual stimuli) were presented using a Sharp LCD projector (29.97 fps). Stimuli were projected onto a translucent plexiglass rear-projection screen mounted on the MRI head coil, in which subjects viewed the stimuli via a head coil mirror. All stimuli were presented using a Macintosh G3 personal computer running MacStim (David Darby, Melbourne, VIC, Australia).

In the scanner, the participants’ instructions were to attend to the presentation of stimuli, and to covertly count instances of a specific target CV token. This orthogonal task was designed to maintain participant attention and compliance. For example, participants were asked to count the number of “ga” stimuli presented during the AV_Cong_ run. Presence of the illusory McGurk perception for these participants was confirmed by repeating the experiment using the same stimuli as presented during the scan on a computer outside of the MR scanner.

### MR IMAGING PARAMETERS

Images were acquired using a 1.5 Tesla Siemens Magnetom Vision whole-body scanner at Georgetown University. Each functional run contained 43 EPI volumes (first 3 pre-stimulus volumes were discarded) that were composed of 25 slices with a slice thickness of 4 mm and a gap of 0.4 mm. We used a repetition time (TR) of 6.5 s, acquisition time (TA) of 3 s, echo time (TE) of 40 ms, and flip angle of 90° with a voxel size of 3.75 mm × 3.75 mm × 4.40 mm. A sparse-sampling design was used to minimize the effect of scanner noise, which is often used in audition studies. EPI volumes were timed to capture the optimal hemodynamic response for each block of trials, allowing the presentation of some stimuli in relative quiet between volumes (Hall et al., 1999). High-resolution MPRAGE scans were acquired using a 256-mm^3^ field of view, with a voxel size of 1.00 mm × 1.00 mm × 1.41 mm. Study design, stimuli, experimental paradigm, MR imaging parameters, and data collection were developed, performed, and published as part of previous work (Zielinski, 2002).

### fMRI DATA ANALYSIS

All statistical tests were performed in 3D volume-space using BrainVoyager QX (*Brain Innovation*) software. MPRAGE and functional images (EPI volumes) were interpolated into Talairach stereotaxic/standard space (Talairach and Tournoux, 1988). Functional images were preprocessed as follows: (1) motion correction using six parameters, (2) temporal high-pass filter including linear trend removal (3 cycles), (3) spatial Gaussian smoothing (6 mm^3^), and (4) co-registration with high-resolution MPRAGE images. During motion correction, images were aligned to the first volume in the run. During spatial normalization, images were aligned across runs. This corrected for any differences in head position both within and across runs.

### WHOLE-BRAIN GROUP ANALYSIS

Whole-brain group analysis was conducted using a fixed-effects general linear model (GLM); the fixed-effects analysis method has been successfully used in the current literature (Leaver et al., 2009; Chevillet et al., 2011). GLM predictors were used to measure changes in fMRI signal in single voxels (Friston et al., 1995) and were defined by the timing of blocks of trials for the four types of experimental conditions: AV_Cong_, AV_McGurk_, A_O_, and V_O_. *Post hoc* contrasts compared AV and unimodal conditions (A_O_ and V_O_) within each fMRI run. Group analyses were corrected for multiple voxel-wise comparisons using cluster thresholds determined by the Monte Carlo method as implemented in Brain Voyager, which estimated the probability of false positives (Forman et al., 1995).

**FIGURE 1:**
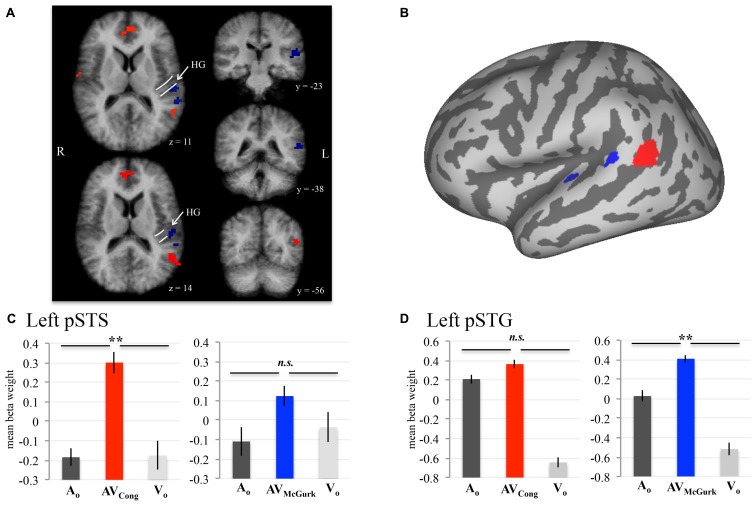
**AV speech areas in the left posterior superior temporal cortex for congruent and McGurk speech.** Group results (*N* = 10; *p*_corr_ < 0.001) showing voxels with significantly higher fMRI signal for AV speech than both types of unimodal speech (acoustic-only, A_O_ and visual-only, V_O_) are displayed on axial (*z* = 11, 14) and coronal (*y* = –23, –38, –56) 3D volume slices of the averaged brain created from the current sample **(A)**. The anatomic designations were determined in 3D volume space relative to the anatomy on the current sample’s averaged brain. The white lines displayed on the axial volume slices approximate the location of HG. Results presented in 3D volume are not interpolated and are presented in radiological convention. The inflated cortical surface template shown in **(B)**, used for display purposes, was not created from the current sample. A conjunction analysis demonstrated that activity in left pSTS (red) was significantly greater in AV_Cong_ trials than in A_O_ and V_O_ trials. Two clusters in left pSTG (blue) exhibited a similar pattern for McGurk speech (i.e., AV_McGurk_ > A_O_ ∩ AV_McGurk_ > V_O_). **(C,D)** Mean fMRI signal for the left pSTS and left pSTG clusters are represented with mean beta weights for AV_Cong_ (red), AV_McGurk_ (blue), A_O_ (dark gray), and V_O_ (light gray) blocks of trials. Beta weights for the left pSTG clusters are first averaged across both clusters in each participant. Error bars denote standard error of the mean across participants, and asterisks (**) mark statistically significant effects in the voxel-wise analysis (*p*_corr_ < 0.001). Abbreviations: HG = Heschl’s gyrus, pSTS = posterior superior temporal sulcus, pSTG = posterior superior temporal gyrus, n.s. = not significant.

To evaluate neural responses to congruent AV speech and McGurk speech across the whole brain, we performed two conjunction (∩) contrasts: (1) AV_Cong_ > A_O_ ∩ AV_Cong_ > V_O_ and (2) AV_McGurk_ > A_O_ ∩ AV_McGurk_ > V_O_ (where both statements flanking ∩ must be true; **Figure 1**; **Table 1**). This type of multisensory comparison corresponds to the “max criterion” method (Beauchamp, 2005b). It is important to note that since no stimulus-absent condition was tested, no statistical comparisons against “rest-baseline” were conducted. Thus, the fMRI signal changes were estimated by relative differences in beta weights. Significant voxels for these conjunction contrasts exhibited greater fMRI signal for the AV condition than for both unimodal conditions (*p*_corr_ < 0.001 and single-voxel threshold *t* > 3.4956, *p* < 0.0005). Whole-brain analyses using Monte Carlo corrections were conducted within a whole-brain mask defined by only those voxels contained within the averaged brain of the current sample (i.e., an average of the skull-stripped MPRAGEs). Mean beta weights and standard errors for each condition are reported across participants for the left pSTS cluster and left pSTG clusters (**Figure 1**). Beta weights for the two left pSTG clusters were averaged first in each participant for every condition, then averaged across participants for the mean beta weight value and standard error. Anatomical location designations of these results were determined based on the anatomy of the averaged brain created from the current sample (*N* = 10) in 3D volume space. These locations were not based on the anatomy of the inflated template cortical surface (**Figure 1B**), which was used only for data presentation and did not reflect the precise anatomy of the current sample.

**Table 1:** Whole-brain group conjunction results (*N* = 10; AV > A_O_ ∩ AV > V_O_) are reported for congruent AV and McGurk speech.

Brain region	Talairach			Volume (mm^3^)
	*X*	*Y*	*Z*	
**Congruent AV speech**
Left pSTS	-53	-56	15	621
Right STG	59	-3	5	459
Medial prefrontal cortex	4	46	9	1998
Cerebellum	-3	-49	-21	432
**McGurk speech**
Left pSTG	-52	-23	12	810
Left pSTG	-57	-38	12	324

*Talairach coordinates represent the center of gravity for each cluster, rounded to the nearest whole number (*p*_corr_ < 0.001).*

### SINGLE-SUBJECT ANALYSIS IN SUPERIOR TEMPORAL CORTEX

**FIGURE 2:**
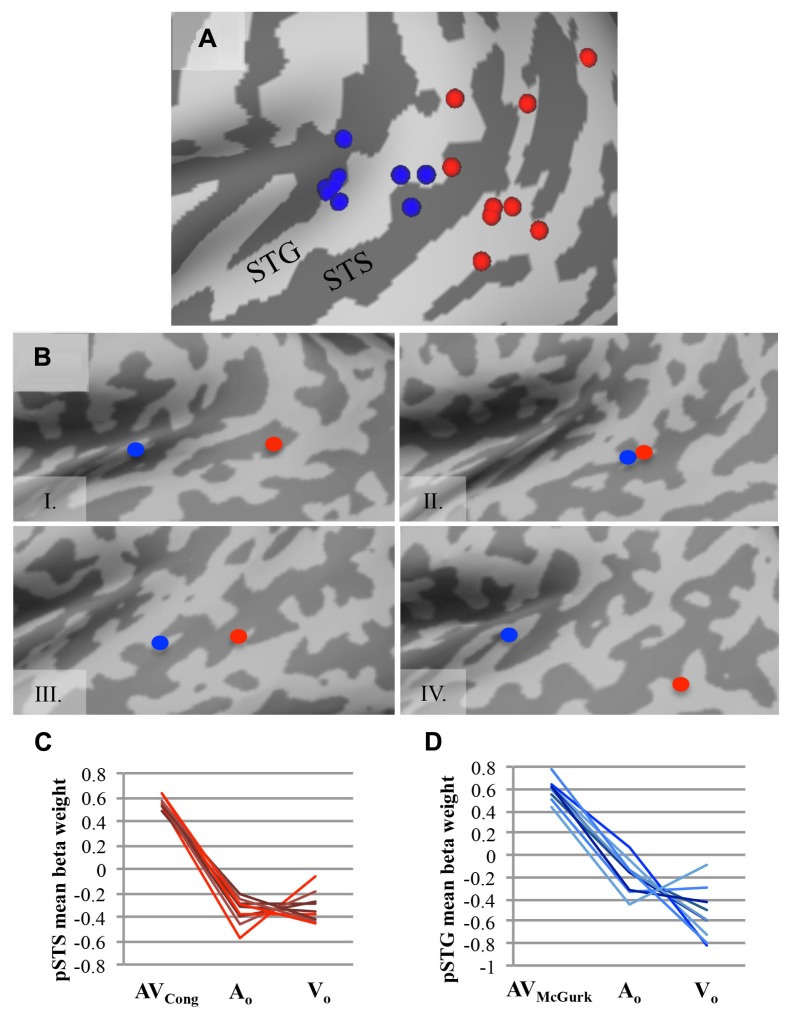
**AV speech areas for congruent and McGurk speech in the left posterior superior temporal cortex: consistency across single subjects. (A)** The center of gravity for clusters with significantly greater fMRI signal for AV trials than for both types of unimodal trials (AV > A_O_ ∩ AV > V_O_) are plotted for each participant on an inflated template cortical surface (single-voxel threshold *t* > 2.2461, *p* < 0.025). Although the functional location varies, nine out of ten participants exhibited a significant AV effect for AV_Cong_ speech in the left pSTS region (red), and all participants but one exhibited significant AV effects for AV_McGurk_ speech in the left pSTG region (blue). **(B)** Four representative single-subject cortical surface maps are displayed with each participant’s foci projected on the inflated surface. Foci represent the center of gravity for clusters with significantly greater fMRI signal for AV speech compared to unimodal speech (AV > A_O_ ∩ AV > V_O_). Red dots represent AV_Cong_ speech, and blue dots represent AV_McGurk_ speech. In most participants, the congruent AV speech clusters were located more posteriorly compared to the McGurk speech clusters. For **(A)** and **(B)**, only the left posterior superior temporal cortex (also including lateral fissure) is displayed. **(C,D)** For each participant, mean fMRI signal from each cluster, estimated by mean beta weight and identified from single-subject activation maps, is depicted for AV and unimodal trials in the left pSTS region **(C)** and the left pSTG region **(D)**; each line represents a single participant. Abbreviations as in **Figure 1**.

Group findings were confirmed using identical contrasts in single-subject analyses (single-voxel threshold *t* > 2.2461, *p* < 0.025; **Figure 2**), because our sample size may not be optimal for random-effects analysis (Petersson et al., 1999a,b), and fixed-effects analysis does not consider subject variability. To identify single-subject activity that best approximated group findings for either congruent AV speech (on or nearby left pSTS) or McGurk speech (on or nearby left pSTG), we selected voxel(s)/cluster(s) significant for each contrast within the left middle to posterior superior temporal cortex on each participant’s brain volume, although other activations (e.g., in temporal cortex) may have been present as well (data not shown). If multiple clusters were chosen for a given subject, then we reported the center of gravity across all clusters together for that participant and mean beta weights were extracted individually from each cluster and averaged for that subject. We validated this selection process by calculating the average Euclidean distance between group and single-subject clusters across participants, using the center of gravity in 3D volume-space.

### “MASKED” ANALYSES RESTRICTED TO SENSORY CORTICES

**FIGURE 3:**
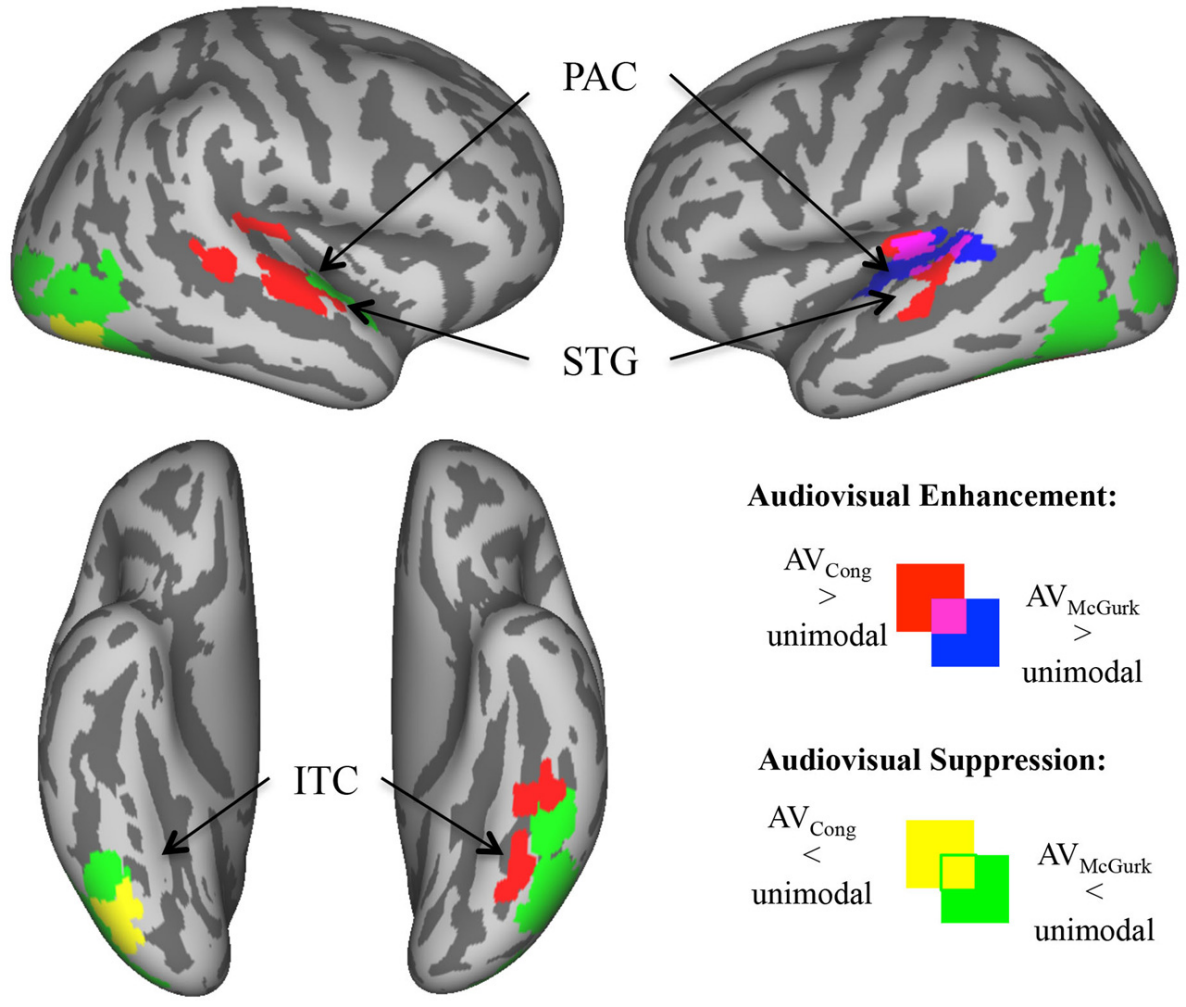
**Enhanced and suppressed activity of sensory cortex by congruent AV and McGurk speech.** In analyses restricted to auditory and visual cortex (via functionally defined masks), voxels exhibiting significantly greater/enhanced or lesser/suppressed activity in AV blocks of trials as compared to A_O_ blocks of trials (in auditory cortex) or V_O_ blocks of trials (in visual cortex) are displayed on inflated template cortical surfaces (*p*_corr_ < 0.01). AV_Cong_ speech (red) compared to unimodal speech had greater fMRI signal in bilateral PAC and mid-STG, and in left ITC including the fusiform gyrus. AV_McGurk_ speech (blue) compared to unimodal speech had greater fMRI signal limited to left PAC and pSTG. By contrast, there were regions of sensory cortex where AV speech had lower fMRI signal compared to unimodal speech. AV_Cong_ speech (yellow) had lower fMRI signal compared to unimodal speech only in right inferior LOC/ITC, whereas during AV_McGurk_ speech (green) stimulation this effect was widely exhibited in bilateral LOC/ITC, and in right ant- to mid-STG. Abbreviations: PAC = primary auditory cortex, ant-STG = anterior superior temporal gyrus, mid-STG = middle superior temporal gyrus, pSTG = posterior superior temporal gyrus, ITC = inferior temporal cortex, LOC = lateral occipital cortex.

To assess neural responses to congruent AV speech and McGurk speech within auditory and visual cortical regions not detected in whole-brain analysis (**Figure 3**), we created auditory and visual cortex masks from within the averaged brain of the current sample. Auditory cortex was defined by a mask within superior temporal lobe that contained voxels surviving either of two conjunction (∩) contrasts: AV_Cong_ > V_O_ ∩ A_O_ > V_O_, or AV_McGurk_ > V_O_ ∩ A_O_ > V_O_. The visual cortex mask was created in a similar way using contrasts: AV_Cong_ > A_O_ ∩ V_O_ > A_O_ and AV_McGurk_ > A_O_ ∩ V_O_ > A_O_. The visual mask included areas within lateral occipital cortex (LOC), and inferior temporal cortex (ITC) containing fusiform gyri. The medial occipital cortex was not included in the mask since A_O_ trials had slightly higher fMRI signal compared to V_O_ trials. This does not preclude medial occipital cortex activation in V_O_ trials; only stimulus-absent trials could confirm this, which were not conducted in this study. To be included in auditory or visual masks, voxels were significant for these contrasts in a whole-brain analysis with a *p*_corr_ < 0.001 determined by single-voxel threshold of *t* > 3.9110, *p* < 0.0001 and displayed with a strict single-voxel threshold of *t* > 5.7940, *p* < 1.0 × 10^-^^8^. AV_Cong_ and AV_McGurk_ effects on masked auditory cortex were defined by two new contrasts: (1) AV_Cong_ > A_O_, and (2) AV_McGurk_ > A_O_ (*p*_corr_ < 0.01; single-voxel threshold *t* > 1.9630, *p* < 0.05). AV_Cong_ and AV_McGurk_ effects on masked visual cortex were defined by two new contrasts: (1) AV_Cong_ > V_O_, and (2) AV_McGurk_ > V_O_ (*p*_corr_ < 0.01; single-voxel threshold *t* > 1.9630, *p* < 0.05). In other words, significant voxels for these contrasts showed greater fMRI signal for AV trials than for auditory (A_O_) trials in masked auditory cortex, or visual (V_O_) trials in masked visual cortex. Notably, the contrasts used to define each sensory cortex mask were different from the contrasts used to investigate the bimodal effects in that sensory cortex mask (Kriegeskorte et al., 2009).

### DATA PRESENTATION

For visualization purposes, group statistics were exported onto an inflated template cortical surface (Van Essen, 2005), using Caret software (Van Essen et al., 2001) or presented on volume slices of the current sample’s averaged brain using BrainVoyager QX (**Figure 1A**). Caret software was used to display foci projections (via “Project Foci to PALS Atlas”) onto an inflated template cortical surface for each single-subject result of statistical tests and corresponding centers of gravity (**Figure 2A**). Additionally, single-subject inflated cortical surfaces were constructed using Freesurfer software (Dale et al., 1999; Fischl et al., 1999). Four representative single-subject results (i.e., center of gravity of single-subject analyses, see sub-section* Single-Subject Analysis*) were projected onto their respective individual inflated cortical surfaces in Freesurfer (“mni2tal”; Brett et al., 2002; **Figure 2B**). One subject’s data resulted in suboptimal surface reconstruction in some cortical areas, but tissue segmentation was accurate in the superior temporal cortex; thus it did not affect the assessment of individual anatomy within this region.

## RESULTS

### BRAIN AREAS INVOLVED IN AV PROCESSING OF CONGRUENT SPEECH

Brain areas associated with processing congruent AV speech were identified from the comparison of the fMRI signal on blocks of trials containing AV recordings of congruent CV stimuli (AV_Cong_) to blocks of trials including only unimodal CV stimuli (A_O_ and V_O_) across the whole brain. The left pSTS exhibited activation where fMRI signal for AV_Cong_ trials was significantly greater than both A_O_ and V_O_ trials (red; **Figure 1**; *p*_corr_ < 0.001 for conjunction contrast: AV_Cong_ > A_O_ ∩ AV_Cong_ > V_O_). Three other brain areas were found: right STG, medial prefrontal cortex, and cerebellum (**Table 1**). In summary, regions identified here, including the left pSTS, have increased response to congruent AV versus unimodal sensory input compared to other areas in the whole brain.

### BRAIN AREAS INVOLVED IN AV PROCESSING OF MCGURK SPEECH

Brain areas involved in processing McGurk speech, composed of incongruent acoustic and visual signals, were identified from the comparison of fMRI signal on blocks of trials containing incongruent McGurk-type AV recordings of CV stimuli (AV_McGurk_) to blocks of trials containing only unimodal CV stimuli (A_O_ and V_O_) across the whole brain (blue; **Figure 1**). Two adjacent clusters were identified in left pSTG, located just posterior to Heschl’s gyrus. It is possible that one of these McGurk clusters may be on the border of Heschl’s gyrus (–52, –23, 12). The anatomical designation of pSTG was based on the anatomy of the current sample’s averaged brain in 3D volume space. These left pSTG clusters exhibited activation where fMRI signal for AV_McGurk_ trials was significantly greater than both A_O_ and V_O_ trials (*p*_corr_ < 0.001 for conjunction contrast: AV_McGurk_ > A_O_ ∩ AV_McGurk_ > V_O_). Increased response to McGurk speech compared to unimodal sensory signals was only identified in regions of the left pSTG.

### SINGLE-SUBJECT CONFIRMATION OF PST REGIONS INVOLVED IN PROCESSING CONGRUENT AV AND MCGURK SPEECH

To confirm the effects found in the group analysis, single-subject analyses were conducted to locate brain areas more responsive to AV_Cong_ or AV_McGurk_ trials compared to unimodal speech, A_O_ and V_O_, using the same statistical contrasts described above. Activation within the left pSTS region was identified for congruent AV speech in nine out of ten participants (**Figure 2**; single-voxel threshold *t* > 2.2461*, p* < 0.025), where the fMRI signal for AV_Cong_ trials was greater than both unimodal trials (A_O_ and V_O_). While the exact location of congruent AV speech clusters identified in the left pSTS region varied among participants, in general, clusters reported here were positioned on the left pSTS or neighboring regions, nearby or overlapping with the group left pSTS finding. These clusters were typically posterior to the individual clusters identified for McGurk speech. However, some participants also showed activation for congruent AV speech in regions similar to the regions identified during McGurk speech (**Figure 2B**). One subject did not show activation to congruent AV speech in left pSTS; however, this subject did show an effect for McGurk speech in left pSTG. The individual locations of congruent AV speech areas differed from the group cluster in the left pSTS by an average of 10.91 ± SD 5.52 mm. The locations of these clusters were carefully determined relative to individual anatomy through evaluations in both volume and in individual surface reconstructions of pST (**Figure 2**).

Recruitment of the left pSTG region was confirmed in processing McGurk speech in single-subject analyses in nine out of ten participants (single-voxel threshold *t* > 2.2461, *p* < 0.025; **Figure 2**), where the fMRI signal for AV_McGurk_ trials was greater than both unimodal trials (A_O_ and V_O_), i.e., using the same conjunction contrast as in the whole-brain group analysis. Individual locations of activation in the pSTG region differed among participants, but in general were positioned on the pSTG or surrounding cortex (e.g., adjacent STS) and were near to or overlapped with the group left pSTG findings. While one participant did not exhibit this effect in left pSTG, this subject did demonstrate the effect in left pSTS for congruent AV speech. The single-subject centers of gravity of fMRI signal compared to the McGurk speech group foci in left pSTG varied by 11.91 ± SD 3.47 mm, averaged for both left pSTG group clusters in each individual, further indicating that there may be individual differences in functional location. Single-subject activations typically overlapped with one or both of the two McGurk group clusters, suggesting that each cluster may likely represent a focal point of activation within the larger area of left pSTG, perhaps extending into Heschl’s gyrus, rather than two areas with distinct functions.

### ENHANCED ACTIVITY IN SENSORY CORTEX BY AV SPEECH

Areas of enhanced activity were localized within masked auditory and visual cortex, where AV blocks of trials exhibited greater fMRI signal compared to unimodal A_O_ blocks of trials in auditory cortex (AV > A_O_) or V_O_ blocks of trials in visual cortex (AV > V_O_). In sensory cortex, congruent AV speech (red; **Figure 3**) had greater fMRI signal compared to unimodal speech bilaterally in primary auditory cortex (PAC) extending into mid-superior temporal gyri (mid-STG), and in left ITC including the fusiform gyrus (*p*_corr_ < 0.01). We consider PAC to be located in medial Heschl’s gyrus (Morosan et al., 2001). In contrast, McGurk speech (blue; **Figure 3**) had greater fMRI signal compared to unimodal speech solely in left PAC spreading into pSTG (*p*_corr_ < 0.01). Overlap of these effects for both congruent AV speech and McGurk speech were localized within the left PAC and pSTG, similar to some single-subject results. In general, these results show that different regions within sensory cortex exhibit preference to congruent AV speech and McGurk speech, complementing results reported above from whole-brain group analyses.

### SUPPRESSED ACTIVITY IN SENSORY CORTEX BY AV SPEECH

Within masked auditory and visual sensory cortex, some regions exhibited significantly lower fMRI signal for AV speech blocks of trials compared to unimodal A_O_ blocks of trials in auditory cortex (AV < A_O_) or V_O_ blocks of trials in visual cortex (AV < V_O_). Activity in these areas of sensory cortex revealed a higher fMRI signal to unimodal speech compared to AV speech. Congruent AV speech (yellow; **Figure 3**) demonstrated lower fMRI signal compared to unimodal trials only in right inferior LOC/ITC (*p*_corr_ < 0.01). This effect was not detected in auditory cortex. In contrast, McGurk speech (green; **Figure 3**) broadly exhibited lower fMRI signal compared to unimodal trials, including right anterior to middle superior temporal gyrus (ant-STG), and bilateral LOC/ITC (*p*_corr_ < 0.01).

## DISCUSSION

Whole-brain group analyses (*N* = 10) that were confirmed in single-subject analyses suggested that distinct posterior superior temporal regions are involved in processing congruent AV and McGurk speech when compared to unimodal speech (acoustic-only and visual-only). Left pSTS was recruited when processing congruent bimodal AV speech, suggesting that this region may be speech-sensitive and critical when sensory signals converge to be compared. In contrast, left pSTG was recruited when processing McGurk speech, suggesting that left pSTG may be necessary when discrepant auditory and visual cues interact. We interpret these findings as suggesting that two similar neural processes take place in separate left pST regions: (1) comparison and integration of sensory cues in the left pSTS and (2) creation of the “corrected” or merged percept in the left pSTG arising from conflicting auditory and visual cues. In other words, a new merged percept is generated in pSTG, resulting from the incorporation of conflicting auditory and visual speech cues. It is possible that alternate interpretations may explain these findings. Future studies will need to more closely examine the precise role of these regions (left pSTG vs. left pSTS) related to general AV-integrative processes. In general, these findings help to support and refine current sensorimotor models of speech processing, especially with regard to multisensory interactions in posterior superior temporal cortex (Skipper et al., 2007; Rauschecker and Scott, 2009; Rauschecker, 2011).

### AV INTEGRATION IN THE LEFT pSTS

The left pSTS was recruited during congruent AV speech, which suggests a general AV-processing function that could support integration of auditory and visual speech signals. The idea that the pSTS is important for multisensory integration (Beauchamp, 2005a; Beauchamp et al., 2008), particularly AV integration of language (Calvert et al., 2000; Beauchamp et al., 2004a; van Atteveldt et al., 2004; Stein and Stanford, 2008; Nath and Beauchamp, 2011) and other stimuli (Beauchamp et al., 2004b; Noesselt et al., 2007; Hein and Knight, 2008; Man et al., 2012; Powers et al., 2012; Watson et al., 2014), is not new. In a recent example, Man et al. (2012) demonstrated similar neural activity patterns in the left pSTS for non-speech visual-only representation and acoustic-only representation of the same object. Supporting our findings, the left pSTS has been consistently recruited in AV language studies using the max criterion for AV integration (conjunction of AV > A_O_ and AV > V_O_; Beauchamp, 2005b) of congruent AV stimuli including various stimulus types, such as sentences in native and non-native language (Barros-Loscertales et al., 2013), words (Szycik et al., 2008), and visual letters paired with speech sounds (van Atteveldt et al., 2004, 2007). Similarly, the left pSTS showed increased activity to congruent AV story stimuli compared to the sum of activity for acoustic-only and visual-only stimulation (Calvert et al., 2000); others have also reported supra-additive AV speech effects in STS (Wright et al., 2003). Evidence that the STS is involved in processing many kinds of sensory input (Hein and Knight, 2008), such as biological motion (Grossman and Blake, 2002) and socially relevant sensory cues (Allison et al., 2000; Lahnakoski et al., 2012), further suggests a general sensory integration function. Our findings and others (Beauchamp et al., 2004a; Man et al., 2012) support the possibility that the pSTS could be responsible for a more general, non-exclusive AV function that compares and integrates AV sensory cues.

Previous studies implicate the left pSTS in the McGurk effect (Sekiyama et al., 2003; Beauchamp et al., 2010; Benoit et al., 2010; Nath et al., 2011; Nath and Beauchamp, 2012). However, these studies do not imply an exclusive role of the left pSTS in the McGurk percept change *per se*. For example, activity in the STS does not always have a strong response to McGurk syllables in some children who have high McGurk percept likelihood (Nath et al., 2011) or a preference to McGurk stimuli over other incongruent AV stimuli in adults (Nath and Beauchamp, 2012). In Japanese speakers, the left pSTS was recruited more during noisy McGurk trials compared to noise-free McGurk trials (Sekiyama et al., 2003), which may reflect an increased demand for AV integration rather than specificity for the McGurk perceptual shift. Further, while inhibitory TMS of the left pSTS significantly decreased the prevalence of reported McGurk percepts, some other AV-influenced percepts were still produced, e.g., “between ‘ba’ and ‘da’,” “b-da,” or new percept “ha,” albeit at a much lower incidence (Beauchamp et al., 2010). This suggests that part of the mechanism responsible for changing or “correcting” the auditory percept based on AV signals is still intact after inactivation of left pSTS. Finally, it is worth noting that left pSTS can be recruited by incongruent (not McGurk stimuli) more than by congruent AV stimuli (Zielinski, 2002; Bernstein et al., 2008; Hocking and Price, 2008; Szycik et al., 2009), perhaps suggesting the left pSTS is involved in situations of incongruence beyond the McGurk effect. Considering our findings in the context of previous work, we suggest that left pSTS may be necessary for the McGurk effect by virtue of its role in general AV processing; however, we suggest the possibility that the resulting change in perception famous to the McGurk effect may occur elsewhere.

### CREATION OF “CORRECTED” PERCEPTS IN THE LEFT pSTG

Our data show that two clusters in the left pSTG (just posterior to Heschl’s gyrus based on the current sample’s averaged brain) were recruited by McGurk speech. One interpretation of our findings is that the left pSTG may have a role in generating new “corrected” percepts underlying the McGurk effect. In other words, pSTG creates a new merged percept by incorporating input from conflicting auditory and visual cues reflective of both streams of information. Previous research, including some McGurk studies, supports this interpretation. One study using pattern analysis in the pSTG and posterior auditory regions was able to decode differences in percept, either “aba” or “ada,” when presented with identical AV stimuli, suggesting that the pSTG is sensitive to perception and not just acoustics (Kilian-Hutten et al., 2011; cf. Chevillet et al., 2013). Despite limited previous evidence, other studies have indicated auditory areas including the pSTG in the McGurk effect (Skipper et al., 2007; Benoit et al., 2010; Szycik et al., 2012), especially where assessments focused on the neural correlates and/or fMRI time courses associated with the change in McGurk speech percept, or the visual modulation present in the McGurk effect. Supporting our findings, Szycik et al. (2012) identified left pSTG activation during McGurk trials when participants reported the McGurk percept and when comparing participants who perceived the McGurk effect to those who did not. Although these pSTG areas are discussed as left “pSTS,” we speculate that it is possible these areas may be on the left pSTG with Talairach foci reported close to the center of gravity of the pSTG clusters identified in our study (our congruent AV pSTS cluster was further posterior). Benoit et al. (2010) showed an adaptation effect for McGurk stimuli in bilateral middle to posterior STG extending into pSTS when the sound was held constant while the visual cue changed, reflecting the auditory perceptual change due to visual influence. Finally, Skipper et al. (2007) provided evidence for percept changes in auditory and somatosensory areas, where early versus late fMRI time courses for McGurk stimuli displayed different neural activation patterns that correlated more to congruent AV “pa” or “ta,” respectively. Building on these previous findings, we propose that, during the McGurk effect, the left pSTG may have a more specific function in generating auditory percepts incorporating the influence of multiple sensory modalities.

### AV ENHANCEMENT AND SUPPRESSION OF ACTIVITY IN SENSORY CORTICES AND OTHER REGIONS

Differential AV responses for congruent AV and McGurk speech are further supported when examining enhancement (increases) and suppression (decreases) of activity in auditory and visual sensory cortex by AV speech compared to acoustic-only or visual-only speech. During congruent AV speech, AV enhancement occurred throughout auditory and visual areas, whereas AV suppression was limited to right LOC. LOC has been previously linked to face/object processing (Grossman and Blake, 2002) and biological motion processing (Vaina et al., 2001). The seeming suppression of the LOC in the right hemisphere in the current study could be related to the left-lateralization of speech/language processes. Similarly, in the main analysis, the right STG had increased activity when comparing congruent AV speech to both acoustic-only and visual-only speech. These results may be due to imagery (Driver, 1996; Kraemer et al., 2005; Zatorre and Halpern, 2005), attention effects (Grady et al., 1997; Pekkola et al., 2006; Tiippana et al., 2011), and/or increased overall input during AV speech compared to only acoustic or visual speech (Hocking and Price, 2008). In contrast, McGurk speech enhancement was only identified in the left pSTG and PAC, and overall there was more AV suppression of auditory and visual sensory cortex. It is possible that the left pSTG and PAC were the only sensory sites benefiting from AV input during McGurk speech, or it could be that these areas process incongruent AV input differently than the rest of sensory cortex. In either case, comparing the relatively widespread enhancement and limited suppression of sensory cortical activity during congruent AV speech to the more circumscribed enhancement of left posterior auditory areas and extensive suppression of sensory cortex during McGurk speech further underscores a potential specialized role of the pSTG in generating auditory percepts reflective of the conflicting AV input present during the McGurk effect.

Although we have focused primarily on the posterior superior temporal cortex, other brain regions are involved in analyzing and integrating AV speech as well. This is exemplified during congruent AV speech, where other regions recruited include medial prefrontal cortex and cerebellum. Medial prefrontal cortex activation has been demonstrated in speech comprehension (Obleser et al., 2007) and recent meta-analytic evidence (Zald et al., 2014) showed consistent coactivation of the adjacent medial and lateral orbitofrontal cortex and the left pST region. The left pSTS and medial prefrontal cortex may process information specific to emotion category (anger, *etc*.), independent of whether the input is received from facial movements, body movements, or the voice (Peelen et al., 2010). Likewise, cerebellum may be involved in speech processing (Sekiyama et al., 2003; Skipper et al., 2005; Ackermann, 2008; Wiersinga-Post et al., 2010), as well as processing music (Leaver et al., 2009). The cerebellum has also been implicated in visual processes related to biological motion, e.g., where biological motion was depicted by visual point-light displays of various human movements (Grossman et al., 2000). Future work is needed to address the interplay and functional relationships between different brain regions during typical AV speech perception. It is important to note that AV interactions not only lead to enhancement of activity; they can also accelerate the detection of visual change in speech, as measured with magnetoencephalography (Möttönen et al., 2002).

### ALTERNATE INTERPRETATIONS AND LIMITATIONS

Alternate interpretations of these findings are possible. For example, AV information may be integrated differently depending on the composition of the AV signal. The processing differences related to integration of McGurk speech could solely result from incongruent auditory and visual sensory inputs and not necessarily from a perceptual change. Similarly, McGurk speech may simply contribute more sensory information than congruent AV speech, where processing of incongruent McGurk speech could have an increased ‘load’ (see Hocking and Price, 2008). However, these interpretations are unlikely because others have found the STS to be activated by McGurk stimuli (Sekiyama et al., 2003; Beauchamp et al., 2010; Benoit et al., 2010; Nath et al., 2011; Nath and Beauchamp, 2012), and other incongruent AV stimuli (Zielinski, 2002; Bernstein et al., 2008; Hocking and Price, 2008; Szycik et al., 2009), suggesting that the STS can process multiple types of AV information including incongruent AV sensory cues. Thus, it is possible that the left pSTG may be involved in a different neural process, such as changing auditory percepts based on the integration of differing auditory and visual cues that are present during McGurk speech. Future experiments are needed to examine bimodal vs. unimodal comparisons with incongruent AV speech stimuli that do not elicit a McGurk or other illusory percepts.

It is also possible that the group findings for McGurk speech in the pSTG extend onto Heschl’s gyrus, because there was variability in the location of the McGurk speech clusters in single-subject analyses, and one of the group McGurk clusters may be on the border of Heschl’s gyrus. The McGurk clusters may overlap with regions equivalent to lateral belt or parabelt areas in non-human primates (Rauschecker et al., 1995; Kaas and Hackett, 2000; Hackett, 2011); however, because these regions are not yet defined with sufficient precision in the human brain (but see Chevillet et al., 2011), the level of auditory processing recruited during McGurk speech is unclear. Thus, if earlier auditory areas including regions of Heschl’s gyrus are recruited during processing of McGurk speech, this would suggest that the “corrected” McGurk percept may be created at an earlier processing stage. Future experiments can further test for perceptual change processes in different regions of the pSTG extending to primary or core auditory areas.

We should note that this experiment also had other limitations. First, while the reported effects in left pSTS and pSTG were identified in whole-brain group analyses and confirmed in single-subject analyses, these results were derived from a relatively small sample (*N* = 10), indicating a slightly lower power than with the standard minimum of *N* = 12 (Desmond and Glover, 2002). Furthermore, the McGurk percept was confirmed in our participants outside of the scanner, in order to limit participant motion, which means the presence of the McGurk effect during the scan is largely inferred. In general, future studies with a larger number of participants are needed to confirm the possibility of differential multisensory effects related to congruent AV speech and the perceptual change associated with the McGurk effect in the pST.

### CONCLUSION: THE MCGURK EFFECT AND THE AUDITORY DORSAL STREAM

Our main findings reveal that the left pSTS may have a more general function in AV processing and the left pSTG may be more involved in processing AV perceptual change. These results have the potential to inform current ideas regarding multisensory function and organization of the pST, particularly in consideration of sensorimotor models of speech processing (Skipper et al., 2007; Rauschecker and Scott, 2009; Rauschecker, 2011). To focus on one model, Rauschecker and Scott (2009) expanded the current dual-stream auditory theory (Rauschecker and Tian, 2000) and proposed that dorsal-stream regions, including the pST, are involved in sensorimotor interactions and multisensory processes. They suggest that these functions may be related to speech and other “doable” sounds, which may facilitate error reduction and “disambiguation of phonological information.” Our findings support this model and further suggest that differential AV interactions within the pST may contribute to these sensorimotor transformations and comparisons. The idea that the McGurk effect may be composed of two neural processes of AV integration and “percept correction,” complements a similar behavioral model, in which the McGurk effect is a two-stage process of “binding and fusion” (Nahorna et al., 2012). In conclusion, we suggest the possibility that the left pSTG and pSTS may have separate functions, wherein the left pSTG may be specially involved in “correcting” incongruent percepts and the left pSTS may function to integrate congruent AV signals.

## AUTHOR CONTRIBUTIONS

All authors meet all four criteria required of authorship. Brandon A. Zielinski and Josef P. Rauschecker conceived of and designed the study; Brandon A. Zielinski, Jennifer E. V. Zielinski, and Guoying Liu conducted data acquisition; Laura C. Erickson, Amber M. Leaver, and Brandon A. Zielinski conducted data analysis; Laura C. Erickson, Brandon A. Zielinski, Peter E. Turkeltaub, Amber M. Leaver, and Josef P. Rauschecker conducted data interpretation; Laura C. Erickson, Amber M. Leaver, Brandon A. Zielinski, and Josef P. Rauschecker wrote the manuscript; all authors critically reviewed the manuscript.

## Conflict of Interest Statement

The authors declare that the research was conducted in the absence of any commercial or financial relationships that could be construed as a potential conflict of interest.
